# Long-Term Mental Health Evaluation After COVID-19: Insights From the CARDIO COVID 20–21 Registry

**DOI:** 10.14740/jocmr6390

**Published:** 2026-01-16

**Authors:** Juan Carlos Rivas Nieto, Brayan Daniel Cordoba-Melo, Juan Pablo Arango-Ibanez, Sebastian Seni-Molina, Mario Miguel Barbosa Rengifo, Carlos Alberto Miranda-Bastidas, Andres Felipe Casanova Rojas, Andres Fernando Mina Sanchez, Cesar J Herrera, Miguel Angel Quintana Da Silva, Andres Felipe Buitrago, Maria Lorena Coronel Gilio, Freddy Pow-Chon-Long, Juan Esteban Gomez-Mesa, on behalf of the CARDIO COVID 20–21 Research Group

**Affiliations:** a Servicio de Psiquiatría, Fundación Valle del Lili, Cali, Colombia; b Departamento de Psiquiatría, Universidad del Valle, Cali, Colombia; c Centro de Investigaciones Clínicas, Fundación Valle del Lili, Cali, Colombia; d Facultad de Ciencias de la Salud, Universidad Icesi, Cali, Colombia; e Hospital Departamental Psiquiátrico Universitario del Valle, Cali, Colombia; f Departamento de Cardiología, Centros de Diagnóstico y Medicina Avanzada y de Conferencias Médicas y Telemedicina (CEDIMAT), Santo Domingo, República Dominicana; g Departamento de Cardiología, Instituto Cardiovascular Sanatorio MIGONE, Asunción, Paraguay; h Departamento de Cardiología, Fundación Santa Fe de Bogotá, Bogotá, Colombia; i Departamento de Cardiología, Instituto de Cardiología Juana Francisca Cabral, Corrientes, Argentina; j Departamento de Cardiología, Hospital Luis Vernaza, Guayaquil, Ecuador; k Servicio de Cardiología, Fundación Valle del Lili, Cali, Colombia

**Keywords:** COVID-19, Long COVID, Depression, Anxiety, Stress, Cognitive dysfunction

## Abstract

**Background:**

Psychopathological manifestations are key features of long COVID, contributing to a considerable global mental health burden. Neuropsychiatric sequelae such as anxiety, depression, cognitive dysfunction, and perceived stress may persist for months or years after infection. Latin American populations remain underrepresented, despite a high prevalence of long COVID and unique socio-demographic characteristics. Understanding these impacts is essential for targeted screening and interventions.

**Methods:**

We conducted a prospective study of patients hospitalized for severe COVID-19. Psychiatric evaluation used the General Anxiety Disorder-7, Patient Health Questionnaire-9, Perceived Stress Scale-14, and Addenbrooke’s Cognitive Examination-III (ACE-III), at an average of 24.5 months post-illness. Bivariate analyses evaluated differences by sex and intensive care unit (ICU) admission. Multivariable linear regression was used to examine associations between cognitive scores and age, sex, education, socioeconomic status, ICU admission, body mass index, smoking exposure, hypertension, and diabetes.

**Results:**

We included 152 patients; the mean age was 56 years, and 58.5% were male. Anxiety symptoms were present in 33%, depression in 49%, and both perceived stress and cognitive dysfunction were each observed in 11% of patients. Women exhibited significantly higher levels of depression (P = 0.02) and stress (P = 0.011), whereas patients admitted to the ICU demonstrated greater cognitive impairment (P < 0.001). In multivariable regression, male sex (P = 0.002), higher education (P < 0.001), and hypertension (P = 0.037) were significantly associated with higher ACE-III scores, while ICU admission was associated with lower scores (P = 0.017).

**Conclusion:**

Our study reveals a high prevalence of mental health symptoms and cognitive dysfunction among patients 2 years after severe COVID-19. Anxiety showed no differences by sex or ICU requirement. Women exhibited higher rates of depression and perceived stress, while ICU admission was associated with poorer cognitive performance. Our findings should encourage systematic screening, diagnosis, and management of long-term neuropsychiatric sequelae in COVID-19 survivors. However, due to the limitations of the single-center design, further longitudinal and multicenter studies are warranted to better elucidate the long-term psychiatric impact of COVID-19.

## Introduction

SARS-CoV-2, the causative pathogen of COVID-19, has brought a major health crisis since 2019 [1]. By 2024, COVID-19 has led to more than 7 million deaths worldwide [2], and its complications go beyond an acute hospitalization episode. In a post-pandemic state, COVID-19 continues to generate problems due to its long-term consequences, representing a major public health challenge [3]. One of the most pressing issues related to this is the profound impact on mental health [4, 5]. The pandemic has led to the emergence of mental disorders as silent yet palpable consequences, calling for a focused examination of these conditions [4, 6].

Psychopathological manifestations in COVID-19 survivors have become key symptoms of “post-COVID condition/syndrome” or, also named, “long COVID”. This topic has become highly relevant given its high incidence and debilitating symptoms [3]. Long COVID is a chronic condition affecting multiple organ systems, characterized by the onset of new or recurring symptoms after the initial acute illness, which can vary in duration and lead to diverse physical, social, and psychological effects [7]. The pathophysiology behind long COVID includes dysfunctional neurological signaling, immune dysregulation, blood clotting and endothelial abnormalities, microbiota dysbiosis, and autoimmunity [3]. Patients with long COVID have an increased risk of sleep disturbances, anxiety, depression, and post-traumatic stress disorder [8–11]. For instance, a recent systematic review and meta-analysis reported pooled estimates of 25% for depression, 23% for anxiety, and 26% for stress among patients with long COVID [12]. Regarding cognitive impairment, although findings are heterogeneous, reported prevalence in the literature ranges from as low as 5% to as high as 50% [13].

It is important to evaluate the neuropsychiatric health of patients who survived COVID-19, as abnormalities could be integral components of long COVID [14]. This is particularly necessary for underrepresented populations in the literature, such as those in Latin America [15], especially since Hispanic patients have an increased prevalence of long COVID [16]. A comprehensive understanding of these aspects is essential for accurate diagnosis and treatment of emerging psychiatric conditions. Furthermore, it facilitates the development of population-targeted strategies by recognizing our population’s specific characteristics, thereby mitigating the impact of such neuropsychiatric sequelae. This study aimed to evaluate the prevalence of anxiety, depression, perceived stress, and cognitive dysfunction 2 years after severe COVID-19 in a Colombian cohort. Additionally, it sought to explore sex-related differences and the impact of intensive care unit (ICU) admission, hypothesizing that mental health symptoms would differ by sex and that ICU admission would be associated with worse cognitive performance, as current literature has suggested differences [3, 17, 18].

## Materials and Methods

### Study design and sample

We conducted a prospective cohort study involving patients who had COVID-19, identified from the CARDIO COVID 19–20 Registry. This database comprised 3,260 hospitalized adult patients hospitalized with microbiologically confirmed COVID-19 between May 1, 2020, and June 30, 2021, from 44 institutions across 14 countries in Latin America [19]. Microbiological diagnosis was established according to World Health Organization criteria, defined as a positive nucleic acid amplification test (NAAT), regardless of clinical or epidemiological criteria, or a positive SARS-CoV-2 antigen test in a person meeting clinical and/or epidemiological criteria. Institutions participating in CARDIO COVID 19–20 were invited to join a follow-up study, CARDIO COVID 20–21 Registry, to assess long-term symptoms, biomarkers, radiological abnormalities, and psychiatric disturbances. Both registries were coordinated and supervised by the Inter-American Council of Heart Failure and Pulmonary Hypertension of the Inter-American Society of Cardiology.

Inclusion criteria for this sub-study included previous severe COVID-19, signing an informed consent, and a complete psychiatric assessment of the scales included in this study. These assessments were conducted at a single participating institution in Colombia, which was the only site performing systematic psychiatric evaluations within the registry.

Severe COVID-19 was defined according to the severity criteria established by the CARDIO COVID 20–21 Registry, which required the presence of at least one of the following during hospitalization: ICU admission; cardiovascular complications (myocardial infarction, heart failure, pulmonary embolism, venous/arterial thrombosis, myocarditis and/or arrhythmias); myocardial injury (elevated troponin); high risk of venous thromboembolism (elevated D-dimer).

These criteria were adapted and endorsed by the study’s scientific committee to ensure methodological consistency across participating sites, as no cardiovascular-specific or universally accepted definition of COVID-19 severity was available at the time the follow-up protocol was developed.

The exclusion criteria for the CARDIO COVID 20–21 Registry included the following: Patients who, at the time of follow-up, had incomplete information in the CARDIO COVID 19-20 database that prevented their contact or in-person visit. Presence of any mental or physical disorder that hindered the performance of clinical or laboratory assessments, or the completion of questionnaires required by the study.

No formal sample size calculation was performed for this subanalysis. A nonprobabilistic convenience sampling method was employed, whereby all patients meeting the predefined inclusion criteria from the parent cohort were included.

This study adhered to the Strengthening the Reporting of Observational Studies in Epidemiology (STROBE) guidelines for reporting observational research.

### Measure and instruments

We assessed patients who agreed to participate between September 2022 and February 2023. Data collection encompassed sociodemographic variables (age, sex, education, among others), clinical variables (comorbidities based on self-report), and psychiatric assessments. The psychiatric evaluation consisted of the assessment of the following scales: General Anxiety Disorder-7 (GAD-7), Patient Health Questionnaire-9 (PHQ-9), Perceived Stress Scale-14 (PSS-14), and Addenbrooke’s Cognitive Examination-III (ACE-III). Psychiatric and cognitive evaluations were conducted during structured in-person encounters by trained research team members, including one psychiatry specialist and two clinical research assistants assigned to the psychiatry service. Cutoff points were established for each scale (GAD-7: > 4, PHQ-9: > 4, PSS-14: > 13, ACE-III: < 88). For the GAD-7 and PHQ-9, these lower thresholds were selected based on prior validation studies showing good sensitivity for detecting mild anxiety and depressive symptoms. These cutoff points allowed differentiation between normal and abnormal results.

These instruments were selected due to their widespread use in clinical and research settings, their ability to evaluate key domains relevant to long COVID-19 mental health burden, including anxiety, depression, stress, and cognitive function, and the availability of validated Spanish versions, including those adapted for Colombian populations. Table 1 summarizes each scale’s utility, diagnostic performance, and classification categories [20–30].

**Table 1:** Psychiatric Scales Assessed

Scale	Use	Categories
GAD-7	A 7-item scale to screen for general anxiety disorder [20]. A cutoff point of 5 has shown a sensitivity of 94% and a specificity of 65% [21]. The version of this scale in Spanish has been previously validated in Colombia [22].	0–4: none to minimal anxiety; 5–9: mild anxiety; 10–14: moderate anxiety; ≥ 15: severe anxiety
PHQ-9	A 9-item scale validated for depression screening [23, 24]. A cutoff point of 5 has a sensitivity of 87% and a specificity of 80% [25]. The version of this scale in Spanish has been previously validated in Colombia [26].	0–4: none to minimal depression; 5–9: mild depression; 10–14: moderate depression; 15–19: fairly severe depression; ≥ 20: severe depression.
PSS-14	A 14-item questionnaire used to measure psychological stress [27]. The Spanish version of this scale has been previously validated [28].	0–13: Low perceived stress; 14–26: moderate perceived stress; 27–40: high perceived stress.
ACE-III	An extended cognitive assessment to screen for cognitive dysfunction. A cutoff score of 88 has a sensitivity of 100% and a specificity of 93% [29]. The Spanish version has been previously validated [30].	100–88: No cognitive dysfunction; 88–82: possible mild cognitive dysfunction; < 82 possible dementia.

ACE-III: Addenbrooke’s Cognitive Examination-III; GAD-7: General Anxiety Disorder-7; PHQ-9: Patient Health Questionnaire-9; PSS-14: Perceived Stress Scale-14.

No other structured psychiatric or cognitive follow-up assessments or interventions were performed by the research team between hospital discharge and the long-term evaluation.

### Statistical methods

Qualitative variables are described using frequency and percentages, while quantitative variables are described using mean and standard deviation (SD) or median and interquartile range (IQR) (when non-normally distributed). The Shapiro-Wilk test was used to assess the data distribution.

For all reported variables in this study, the proportion of missing data was below 5%. Given the minimal extent of missingness, no imputation methods were applied, and analyses were conducted using complete case data only.

We conducted a bivariate analysis to assess differences between participants by sex and history of ICU admission during acute COVID-19. The Chi-square test, exact Fisher’s test, and Kruskal-Wallis test were used to compare these groups. Bivariate analyses were conducted to comprehensively characterize the baseline characteristics of the study population and to assess differences by sex and ICU admission status across all implemented scales.

We conducted a multivariable linear regression to assess factors associated with ACE-III score. Independent variables included age, sex, years of education, socioeconomic status, ICU admission, body mass index (BMI), smoking exposure, hypertension, and diabetes mellitus. These variables were selected *a priori* based on existing evidence linking them to cognitive outcomes in long COVID, critical illness, and broader literature on modifiable and non-modifiable cognitive risk factors. Age, sex, education, and socioeconomic status are established determinants of baseline cognitive performance, while ICU admission reflects disease severity and has been associated with long-term neurocognitive impairment. Smoking, BMI, hypertension, and diabetes have also been linked to cognitive decline in various populations. For each adjusted linear regression model, we assessed homoscedasticity, normal distribution of residuals, absence of multicollinearity, and absence of extreme values. The normal distribution of residuals was determined through visual inspection of the histogram.

Further statistical analysis was conducted to evaluate the variance inflation factors (VIFs) for the predictors in the regression model, aiming to confirm the absence of multicollinearity. The obtained VIF values remained below the critical threshold of 10, indicating the lack of significant multicollinearity among the variables. The residual plots and influence diagnostics suggest that the linear regression model’s assumptions were satisfied. The influence plot and the influence index plot were employed to identify potential outliers and influential observations within the regression model. These diagnostic procedures corroborate that most observations adhere to the assumptions of the linear regression model.

The statistical analysis was performed using R software version 2023.12.0+369. Figures were created using R software version 2023.12.0+369, Seaborn library, and Lucidchart.

### Ethical considerations

This study received approval from the Comité de ética en Investigación Biomédica (protocol 1756, approved on 21 April 2021), the institutional review board of Fundación Valle del Lili. All patients signed an informed consent. The objectives of the study, as well as the benefits and rights of participants, were explained to them. This study complies with the principles outlined in the Declaration of Helsinki.

Artificial intelligence-assisted technologies were used solely to improve the clarity and readability of the manuscript. These tools were not involved in the generation, analysis, or interpretation of the data.

## Results

**Figure 1:**
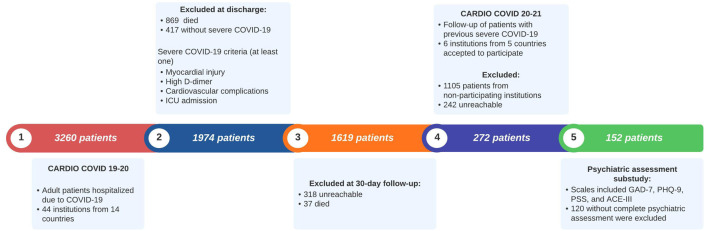
Flowchart of patient selection for the CARDIO COVID 20–21 psychiatric assessment. ACE-III: Addenbrooke’s Cognitive Examination; GAD-7: General Anxiety Disorder-7; ICU: intensive care unit; PHQ-9: Patient Health Questionnaire-9; PSS-14: Perceived Stress Scale-14.

The CARDIO COVID 19–20 Registry included 3,260 patients. During hospitalization, 869 died, 417 did not meet the criteria for severe COVID-19, 37 died at a 1-month follow-up, and 318 were unreachable at the 1-month follow-up. For the CARDIO COVID 20–21 Registry, six institutions from five countries (Argentina, Colombia, Ecuador, the Dominican Republic, and Paraguay) participated. Out of the 1,619 initially eligible patients, 1,105 were excluded as they were evaluated at non-participating institutions. Of the remaining 514 patients, 242 were excluded due to being unreachable or declining participation. A total of 272 patients agreed to participate. For this subanalysis, we included 152 patients with complete psychiatric assessments, which were conducted in one participating institution from Colombia as it was the only one conducting these assessments (Fig. 1).

Baseline characteristics of the 152 patients are summarized in Table 2. The mean age was 56 years, and 58.5% were men. ICU admission occurred in 57% of participants, being more frequent among men (67%) than women (33%). The median ICU stay was 10 days (IQR 5–19). Among female patients, the median was 10 days (IQR 2–21), and among male patients, 10 days (IQR 6–17). There was no statistically significant difference between sexes (P = 0.653). The average follow-up time after the acute event was 24.5 months, with no differences by sex or ICU admission. Patients admitted to the ICU had fewer years of education compared with those not admitted (mean 9.9 vs. 11.7; P = 0.012), while age and socioeconomic status were similar across groups.

**Table 2:** Baseline Characteristics Stratified by Sex and ICU Admission

Variable	All, N = 152	Sex			ICU admission		
		Female, N = 63	Male, N = 89	P value	No, N = 64	Yes, N = 88	P value
Demographics							
Age, years^a^	56 (14)	54 (16)	57 (12)	0.3	54 (16)	57 (13)	0.15
Years of education^a^	10.7 (4.8)	10.7 (4.4)	10.6 (5.1)	0.8	11.7 (4.2)	9.9 (5.0)	0.012
Socioeconomic status^a,b^	2.64 (1.37)	2.54 (1.39)	2.71 (1.36)	0.374	2.83 (1.55)	2.50 (1.21)	0.316
Follow-up duration (months)^a^	24.5 (2.05)	24.4 (2.21)	24.6 (1.94)	0.7	24.8 (1.80)	24.4 (2.21)	0.3
Comorbidities							
Overweight/obesity	74 (48%)	32 (50%)	42 (49%)	0.9	26 (41%)	48 (56%)	0.10
Hypertension	62 (40%)	26 (41%)	36 (40%)	> 0.9	26 (40%)	36 (40%)	> 0.9
Diabetes mellitus	26 (17%)	10 (16%)	16 (18%)	0.8	2 (3.2%)	24 (27%)	< 0.001
Pharmacological history							
ARB	50 (33%)	20 (32%)	30 (34%)	0.80	22 (34%)	28 (32%)	0.74
Statins	34 (22%)	14 (22%)	20 (22%)	0.97	10 (16%)	24 (27%)	0.089
Hypoglycemic drugs	28 (18%)	13 (21%)	15 (17%)	0.55	5 (7.8%)	23 (26%)	0.004
Physical examination and lifestyle							
SBP (mm Hg)^a^	127 (15)	127 (15)	127 (16)	0.994	125 (16)	128 (15)	0.217
DBP (mm Hg)^a^	78 (9)	77 (9)	79 (9)	0.108	79 (9)	78 (9)	0.833
Heart rate (bpm)^a^	72 (12)	74 (12)	71 (11)	0.109	72 (11)	73 (12)	0.575
BMI (kg/m^2^)^a^	28.5 (4.9)	29.1 (5.2)	28.2 (4.7)	0.426	27.5 (4.2)	29.3 (5.2)	0.029
Smoking exposure^c^	21 (14%)	6 (9.5%)	15 (17%)	0.197	7 (11%)	14 (16%)	0.380

Statistical significance was evaluated using Fisher’s exact test, Pearson’s Chi-squared test, or Wilcoxon rank-sum test, as appropriate. ^a^Values are presented as mean ± standard deviation. ^b^In Colombia, residential areas are classified into six socioeconomic strata (1–6) based on income levels. Stratum 1 represents the lowest-income areas receiving the greatest public utility subsidies, whereas Stratum 6 corresponds to the highest-income areas paying full rates without subsidies. ^c^Smoking exposure refers to previous or current smoking. ARB: angiotensin receptor blocker; BMI: body mass index; DBP: diastolic blood pressure; ICU: intensive care unit; SBB: systolic blood pressure.

Overweight or obesity was the most prevalent comorbidity (48%), followed by hypertension (40%) and diabetes mellitus (17%). Diabetes and the use of hypoglycemic agents were more frequent among patients admitted to the ICU compared with those not requiring intensive care (P < 0.001 and P = 0.004, respectively). Mean BMI was higher among ICU-admitted patients than among non-ICU patients (29.3 vs. 27.5 kg/m^2^; P = 0.029), whereas blood pressure, heart rate, and smoking exposure did not differ across groups. No sex-based differences were observed in clinical or pharmacological variables. Other baseline characteristics, comorbidities, and pharmacological treatments of lower frequency are presented in Supplementary Materials 1 and 2 (jocmr.elmerjournals.com).

Table 3 summarizes the results of the GAD-7, PHQ-9, PSS-14, and ACE-III scales by sex and ICU admission. In the GAD-7 assessment, 33% had abnormal scores, most of which fell within the “mild” category (20%). No significant differences were observed regarding abnormal scores between women and men (41% vs. 27%, respectively, P = 0.080) or ICU admission (31% vs. 34%, P = 0.07).

**Table 3:** GAD-7, PHQ-9, PSS, and ACE-III by Sex and ICU Admission

Scale	All, N = 152	Sex			ICU admission		
		Female, N = 63	Male, N = 89	P value	No, N = 64	Yes, N = 88	P value
GAD-7				0.052			0.7
No symptoms or minimal	102 (67%)	37 (59%)	65 (73%)		44 (69%)	58 (66%)	
Mild symptoms	30 (20%)	14 (22%)	16 (18%)		10 (16%)	20 (23%)	
Moderate symptoms	18 (12%)	12 (19%)	6 (6.7%)		9 (14%)	9 (10%)	
Severe symptoms	2 (1.3%)	0 (0%)	2 (2.2%)		1 (1.6%)	1 (1.1%)	
Abnormal (> 4)	50 (33%)	26 (41%)	24 (27%)	0.08	20 (31%)	30 (34%)	0.07
PHQ-9				0.02			0.8
None-minimal	78 (51%)	25 (40%)	53 (60%)		31 (48%)	47 (53%)	
Mild	46 (30%)	21 (33%)	25 (28%)		21 (33%)	25 (28%)	
Moderate	16 (11%)	8 (13%)	8 (8.9%)		7 (11%)	9 (10%)	
Moderate-severe	11 (7.2%)	9 (14%)	2 (2.2%)		4 (6.3%)	7 (8%)	
Severe	1 (0.7%)	0 (0%)	1 (1.1%)		1 (1.6%)	0 (0%)	
Abnormal (> 4)	74 (49%)	38 (60%)	36 (40%)	0.021	33 (52%)	41 (47%)	0.6
PSS-14				0.011			0.5
Low perceived stress	136 (89%)	51 (81%)	85 (95.5%)		57 (89%)	79 (89.8%)	
Moderate perceived stress	12 (7.9%)	9 (14%)	3 (3.4%)		6 (9.4%)	6 (6.8%)	
High perceived stress	4 (2.6%)	3 (4.8%)	1 (1.1%)		1 (1.6%)	3 (3.4%)	
Abnormal (> 13)	16 (11%)	12 (19%)	4 (4.5%)	0.006	7 (11%)	9 (10.2%)	> 0.9
ACE-III				0.018			0.007
No cognitive dysfunction	135 (89%)	52 (83%)	83 (93.3%)		63 (98.4%)	72 (82%)	
Cognitive dysfunction (< 88)	17 (11%)	11 (17%)	6 (6.7%)	0.065	1 (1.6%)	16 (18%)	< 0.001

Statistical significance was evaluated using Wilcoxon rank sum test and Fisher’s exact test for count data; Fisher’s exact test for count data with simulated P-value (based on 2,000 replicates) was used. ACE-III: Addenbrooke’s Cognitive Examination; GAD-7: General Anxiety Disorder-7; ICU: intensive care unit; PHQ-9: Patient Health Questionnaire-9; PSS-14: Perceived Stress Scale-14.

In the PHQ-9, 51% of participants were classified as “none-minimal,” while the majority of those with abnormal scores (49%) were categorized as having mild signs of depression (30%). A significant difference was observed between women and men (60% vs. 40%, P = 0.021), but no difference was found by ICU admission history (52% vs. 47%, P = 0.6).

In the PSS-14 scale, 89% of participants were categorized as having “low perceived stress.” Of the patients, 11% were found to have a significant perception of stress; most had moderate perceived stress (7.6%). A significant difference was observed between women and men (19% vs. 4.5%, P = 0.006), but no difference was found when stratifying by ICU admission (11% vs. 10.2%, P > 0.9).

Median ACE-III was 96 points, with a statistically significant difference between women and men (P = 0.018) and between the group that went to the ICU and those who did not (P = 0.007). Cognitive impairment was seen in 11% of patients (score less than 88), and a statistically significant difference was found between the group that went to the ICU and those who did not (18% vs. 1.6%, P = 0.001).

**Figure 2:**
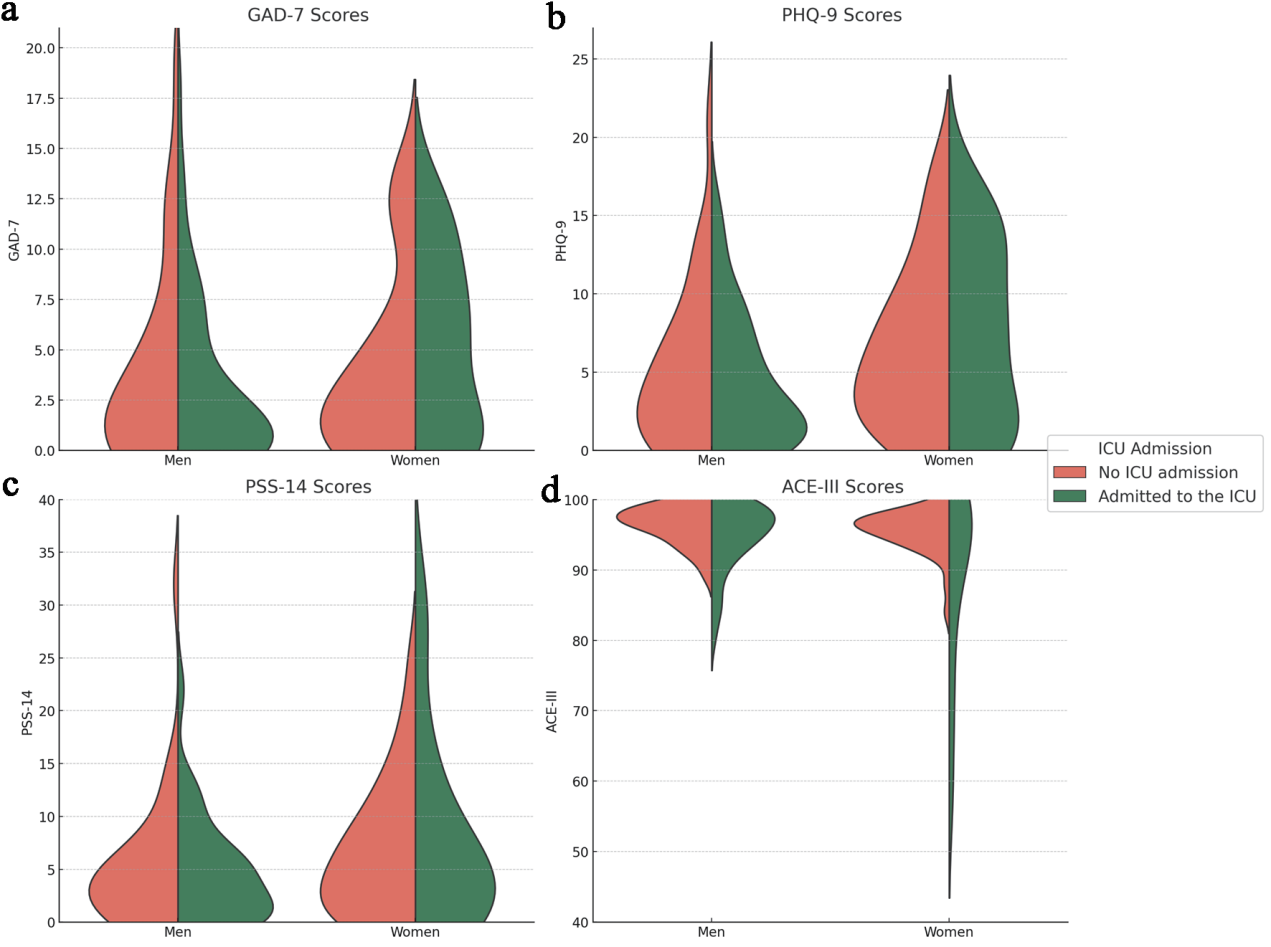
Violin plots of psychiatric scales by sex and ICU admission: (a) GAD-7 scores; (b) PHQ-9 score; (c) PSS-14 score; (d) ACE-III score. Violins are stratified by sex (men and women), and split by ICU admission. Orange shading represents data on patients who did not require ICU admission, while green shading represents data on patients admitted to the ICU. ACE-III: Addenbrooke’s Cognitive Examination; GAD-7: General Anxiety Disorder-7; ICU: intensive care unit; PHQ-9: Patient Health Questionnaire-9; PSS-14: Perceived Stress Scale-14.

Figure 2 displays violin plots of the psychiatric scales, stratified by sex and split by ICU admission status. The GAD-7 graph (Fig. 2a) illustrates a tendency toward higher density of moderate-to-severe anxiety scores among women compared to men, particularly those admitted to the ICU, though men appeared to reach higher peak scores. This pattern is also evident in the PHQ-9 graph (Fig. 2b), with a more pronounced density difference in moderate-to-severe scores. PSS-14 scores (Fig. 2c) displayed a similar distribution pattern. The ACE-III graph (Fig. 2d) reveals higher density of lower scores among ICU-admitted patients, with women reaching the lowest cognitive scores.

**Figure 3:**
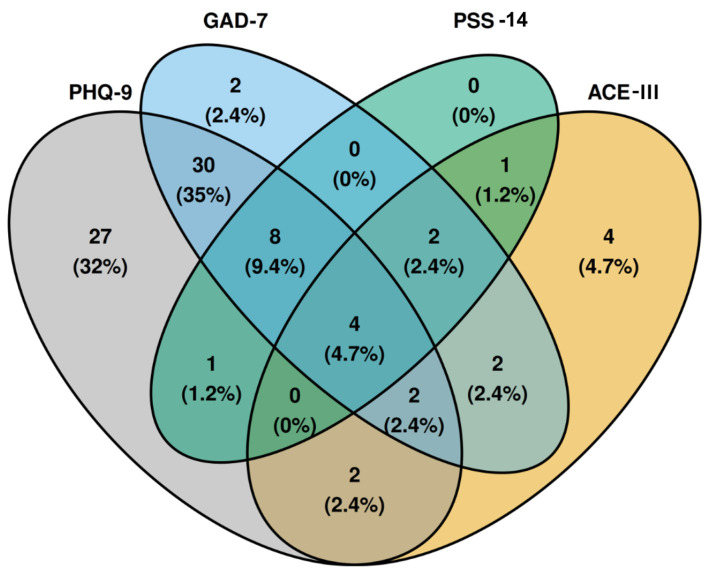
Four-dimensional Venn diagram demonstrating the co-occurrence of abnormal scores between scales. Percentages are calculated based on the total number of abnormal scores. ACE-III: Addenbrooke’s Cognitive Examination-III; GAD-7: General Anxiety Disorder-7; PHQ-9: Patient Health Questionnaire-9; PSS-14: Perceived Stress Scale-14.

Figure 3 is a four-dimensional Venn diagram demonstrating the co-occurrence of abnormal scores between scales. Abnormal scores on any scale occurred in 85 (56%) patients. A high co-occurrence of an abnormal PHQ-9 and GAD-7 score is seen in 30 patients (35% of abnormal scores). Eight (9.3%) patients have an abnormal PHQ-9, GAD-7, and PSS-14, with the highest percentage of co-occurrence in more than two scales.

Given the high co-occurrence of abnormal scores on the GAD-7 and PHQ-9, we performed a linear regression analysis with GAD-7 as the independent variable. This analysis revealed a positive correlation with a correlation coefficient of 0.708. Additionally, we observed an R^2^ of 0.502 and a beta (β) coefficient for GAD-7 of 0.805 (P < 0.001).

The results of the multivariable linear regression model of ACE-III scores are presented in Table 4. In this model (R^2^ = 0.21, adjusted R^2^ = 0.16; σ = 6.6), years of education, male sex, admission to the ICU, and hypertension were independently associated with ACE-III scores. Each additional year of education was associated with a 0.47-point increase in ACE-III score (β = 0.47; 95% CI 0.20–0.74; P < 0.001), male sex was associated with a 3.66-point higher score compared with female sex (β = 3.66; 95% CI 1.40–5.93; P = 0.002), and hypertension with a 2.56-point higher score (β = 2.56; 95% CI 0.16–4.96; P = 0.037). ICU admission was associated with a 2.95-point lower score (β = −2.95; 95% CI −5.37 to −0.54; P = 0.017). Other variables, including age, diabetes, BMI, smoking, and socioeconomic status, were not significantly associated with ACE-III scores. Model diagnostics indicated no relevant multicollinearity (All VIFs were below 2) and confirmed linearity, homoscedasticity, and normal distribution of residuals.

**Table 4:** Univariable and Multivariable Linear Regression Analysis of ACE-III

Variable	Univariable			Multivariable		
	Beta	95% CI	P value	Beta	95% CI	P value
Age	−0.05	−0.13, 0.03	0.23	−0.004	−0.10, 0.09	0.94
Male	2.96	0.65, 5.27	0.01	3.66	1.40, 5.93	0.002
Years of education	0.49	0.26, 0.72	< 0.001	0.47	0.20, 0.74	< 0.001
ICU admission	−3.10	−5.40, −0.80	0.01	−2.95	−5.37, −0.54	0.02
BMI (kg/m^2^)	−0.01	−0.25, 0.23	0.92	0.02	−0.21, 0.26	0.84
Smoking exposure^a^	−1.51	−4.88, 1.85	0.38	−1.75	−5.04, 1.53	0.29
Hypertension	0.84	−1.49, 3.17	0.48	2.56	0.16, 4.96	0.04
Diabetes mellitus	−1.38	−4.01, 1.25	0.30	−0.87	−3.61, 1.86	0.53
Socioeconomic status	0.63	−0.22, 1.48	0.14	−0.10	−0.98, 0.77	0.82

^a^Smoking exposure refers to previous or current smoking. ^b^Socioeconomic status was based on Colombia’s official residential socioeconomic strata classification, which categorizes households into six levels (1–6) according to housing and neighborhood characteristics. ACE-III: Addenbrooke’s Cognitive Examination III; BMI: body mass index; CI: confidence interval; ICU: intensive care unit.

## Discussion

This study provides a comprehensive analysis of the mental health of patients who experienced severe COVID-19 in a Colombian population at a 2-year follow-up. Significant rates of mental health disorders were observed, with anxiety present in 33% of patients, depression in 49%, as well as perceived stress and cognitive impairment in 11%. We also found significant differences in long-term mental health outcomes between women and men, as well as between ICU-admitted and non-ICU patients.

We found a high prevalence (33%) of anxiety symptoms in our cohort, a finding that is lower than those from a meta-analysis showing a pooled prevalence of anxiety in COVID-19 patients of 47% (95% CI 37–57%, I^2^ = 97%) [8]. In contrast, a meta-analysis on anxiety in long COVID patients, found a pooled prevalence of 23% (95% CI 20–26%; I^2^ = 99.9%), a finding closer to ours [31]. Differences in population and methodological characteristics, reflected in the high heterogeneity across studies (I^2^ > 97–99%), likely explain the variability in reported prevalence and why our findings fall within the range of existing meta-analyses, without indicating a truly different anxiety burden. A study following COVID-19 survivors for 24 months, found a prevalence of anxiety of 24.2% [32]. Likely, the actual proportion of patients experiencing an anxiety disorder in our cohort is lower, as the diagnostic accuracy of the GAD-7 using a cutoff of 5 or greater is inferior to other cutoffs, such as 8 [21]. Thus, the proportion would be closer to that of the study following patients at 24 months, the same follow-up period intended in our study. Even with more conservative estimates in our cohort, the proportion appears higher than the estimated lifetime prevalence of any anxiety disorder in the Colombian population (3.9%, 95% CI 3.4–4.6) [33]. Neither sex nor ICU admission demonstrated significant differences in anxiety symptoms, suggesting more uniform manifestation across severe COVID survivors. While evidence indicates that one-third of ICU survivors experience persistent anxiety, that same systematic review found no gender association with anxiety [34], consistent with our findings despite our broader cohort including both ICU and non-ICU patients. This may reflect the pandemic’s widespread psychological impact along with methodological variations such as different assessment instruments.

Our study revealed a high proportion of patients experiencing significant levels of depressive symptoms through the PHQ-9, suggesting a high prevalence of depression (49%). These levels are considerably higher than those observed in hospitalized patients (5–34%) and outpatients (27%) before the pandemic [35, 36]. On the other hand, this estimate is highly comparable to the findings reported by a meta-analysis on the prevalence of depression in COVID-19 patients, which indicated a prevalence of 45% (95% CI: 37–54%, I^2^ = 96%); this study used the same PHQ-9 threshold that we used to diagnose depression [8]. However, a PHQ-9 cutoff score of 4 tends to overestimate the prevalence of depression, which could explain the slightly higher prevalence in our cohort. Therefore, using a threshold of 10 (equal or greater), which has better diagnostic accuracy, provides a more accurate estimate of depression [37]. With this higher cutoff, the overall prevalence of depression in our cohort is 18.9%. This prevalence is still considerably higher than the lifetime prevalence of depressive-related conditions (major depression, minor depression, and dysthymia) in Colombia, which is estimated to be 5.4% (95% CI 4.7–6.2) [33].

The adjusted prevalence of depression in our cohort (18.9%) is closer to that reported in the literature. A meta-analysis of patients with long COVID estimated a prevalence of 23% (95% CI: 20–26%; I^2^ = 99.9%) [31]. A study including 1,276 COVID-19 survivors conducted a 12-month follow-up and found a prevalence of depression or anxiety at 26%. Additionally, they reported an increase in this prevalence compared to the 6-month visit, which was 23% [38, 39]. Another study evaluated patients 24 months after acute COVID-19 and found a prevalence of depression of 19.7%—very similar to ours, although the authors measured it using the EuroQol-5 Dimension Index [32]. Depression rates were significantly higher in women than men, a finding consistent with a meta-analysis indicating that women have a higher risk of depression among long COVID patients [40].

Besides a high prevalence of both anxiety and depression, we found a high co-occurrence of these two (32% of patients with any abnormal scores). This is also supported by a positive correlation between higher GAD-7 and PHQ-9 scores (correlation coefficient 0.708). This significant co-occurrence has already been studied in the literature and the reported prevalence is substantially high [41]. In the context of COVID-19, several studies have reported similar associations. A study involving 1,142 patients with previous COVID-19 inpatient treatment found a positive correlation between symptoms of anxiety and depression, with a correlation coefficient of 0.759 (P < 0.001) [42]. A prospective study reported a strong positive relationship between anxiety symptoms and depression severity, with a Spearman’s rank correlation coefficient of 0.716 (P < 0.001) [43]. The high prevalence of anxiety and depression among COVID-19 survivors is multifactorial and could be explained by long COVID, multimorbidity, and pandemic-related challenges, among many other factors [31, 44, 45].

Regarding PSS-14, 11% of patients experienced moderate or high perceived stress. Few studies have examined perceived stress in recovered patients. One study found that 53% of patients had moderate to severe stress, and 47% mild stress at 3 months follow-up [46]. The longer duration between illness and evaluation might explain the lower percentage observed in our study. In Colombia, during the height of the COVID-19 pandemic in 2020, a study on the general population reported that 15% of participants presented high perceived stress levels [47]. Our findings reveal a similar prevalence of high and moderate perceived stress levels even years after the pandemic peak, suggesting a lasting impact on mental health and the enduring consequences of the pandemic. Additionally, women showed significantly higher perceived stress levels than men, aligning with studies suggesting that they are at increased risk for stress disorders [48].

Several studies have focused on post-traumatic stress disorder (PTSD) and COVID-19. A meta-analysis found that 18% of patients experienced PTSD after discharge, likely due to hospitalization and isolation [49]. Notable percentages of probable, partial, or subthreshold PTSD have also been reported, indicating considerable distress [48, 50]. This raises concerns, in our cohort of patients, about potential residual PTSD symptoms beyond 2 years post-infection.

Importantly, both depression and perceived stress were affected by sex but not by ICU admission. This pattern suggests that sex-related biological, psychological, or social factors may play a more significant role than ICU requirements in shaping long-term depressive and stress responses. It also highlights the need for sex-specific interventions in post-COVID mental health care and further investigation into the mechanisms driving these disparities.

The ACE-III assessment revealed that 11% of patients had cognitive impairment. Current literature supports a long-term association between cognitive decline and prior COVID-19 infection. A meta-analysis concluded that approximately one-third of patients with a previous COVID-19 diagnosis experienced cognitive impairment over a follow-up period of more than 12 weeks [51]. Another study with 1,487,712 patients demonstrated that patients with a previous COVID-19 infection had a hazard ratio of 1.36 for cognitive impairment at the end of a 2-year follow-up period [52]. Aziz et al reported that 46% of a cohort of patients, with a median age of 44.5 years and a median follow-up of 10.4 months from COVID-19, exhibited mild cognitive dysfunction. The study employed the Montreal Cognitive Assessment, which thoroughly evaluates cognitive function [53]. In Colombia, a survey by the Ministry of Health reported a prevalence of 8.9% for cognitive impairment without dementia in a population with a median age of 70.8 years [33]. Interestingly, our study found a higher prevalence of cognitive deficits in a younger population, despite the established increased risk of older age [54].

Through a multivariable model, ICU admission remained associated with lower ACE-III scores, confirming the bivariate findings and underscoring a potential connection between ICU-level severity and long-term cognitive impairment. Other studies support this relationship. For example, in a study involving 141,583 participants—282 admitted to the ICU—the researchers used a propensity score to identify an association between ICU-admitted COVID-19 patients and greater cognitive differences compared to patients without COVID-19 [55]. Another study with 213 participants found that ICU-admitted COVID-19 patients exhibited more severe long-term cognitive impairment [17]. A study found that patients with severe symptoms who were admitted to the ICU were more susceptible to developing cognitive impairment compared to those with moderate and mild symptoms at a 4-month follow-up [56]. Our statistical approach, which allowed us to isolate the unique contribution of ICU admission through multivariable adjustment, together with the consistency of our findings with existing literature, reinforces its role as a severity-related factor associated with long-term cognitive impairment in survivors of severe COVID-19.

The association between ICU admission and cognitive impairment may reflect not only the impact of severe COVID-19, but also the effects of intensive care itself. This pattern is consistent with post-intensive care syndrome (PICS), a well-established condition involving persistent cognitive, psychological, and physical impairments after ICU stay [57]. A recent meta-analysis confirmed that cognitive impairment persists in over 30% of ICU survivors beyond 12 months, with no significant differences between COVID-19 and non-COVID-19 populations [58]. Additional PICS-related factors, such as financial strain and family disruption, may have contributed to the findings observed, especially given the long follow-up period [57, 59]. This convergence between PICS and post-COVID cognitive sequelae reinforces the need to consider ICU-related factors when interpreting long-term neurocognitive outcomes.

Additionally, although bivariate analyses showed no significant sex differences in cognitive performance, male sex emerged as a protective factor in the multivariable model. This suggests a meaningful sex-based vulnerability, which is consistent with literature indicating that females may be more affected by cognitive dysfunction after COVID-19 [51]. Alongside sex differences in depression and stress, these findings suggest greater female vulnerability to the cognitive and psychological effects of severe COVID-19. In addition, years of education, a well-established protective factor for cognitive performance, and hypertension were both associated with higher ACE-III scores after adjustment, the latter being consistent with evidence that antihypertensive treatment may reduce cognitive decline [60].

The use of validated psychiatric scales strengthens the reliability of our findings, as all instruments employed have been validated in Spanish. The selection of these complementary instruments enabled a comprehensive assessment across mental health domains. The PHQ-9 and GAD-7 are brief, widely used screening tools with strong psychometric properties for detecting symptoms of major depression and generalized anxiety, respectively. Their brevity facilitates large-scale assessment, but as screening tools, they assess symptom severity rather than offering full diagnostic characterization. Furthermore, their specificity to these disorders may not capture other relevant conditions, such as bipolar disorder or social anxiety, which could influence long-term outcomes. The PSS-14 assesses global perceived stress, though it lacks diagnostic specificity and may conceptually overlap with depressive and anxiety symptoms. The ACE-III provides detailed screening across multiple cognitive domains, but its performance can be influenced by educational attainment and cultural background, particularly in heterogeneous populations.

This study presents a comprehensive evaluation of long-term mental health and cognitive outcomes following severe COVID-19 in a Latin American country. Using validated instruments to assess anxiety, depression, perceived stress, and cognition, our analysis provides a multidimensional and methodologically rigorous perspective on long COVID neuropsychiatric sequelae in an underrepresented population. The associations identified between sex and ICU admission with mental health outcomes suggest that both biological and contextual factors may influence long-term trajectories. The persistence of symptoms 2 years after infection underscores the chronic nature of this condition and highlights the need for sustained and multidisciplinary care beyond the acute phase. This includes psychiatric and cognitive screening, multidisciplinary follow-up programs, and targeted interventions for high-risk groups, such as women and ICU survivors.

### Strengths and limitations

This study’s strengths rely on its prospective nature and broad psychiatric assessment. Evaluations were conducted in person, in contrast to other studies that evaluated patients virtually. By selecting hospitalized patients who experienced severe COVID-19, we focused on a specific population with greater disease severity, allowing for the evaluation of a potential relationship between disease severity and mental health outcomes. Other studies have not focused on the baseline characteristics of the disease. Additionally, the 2-year follow-up period enables the observation of the long-term nature of this potential association. A further strength is the use of a multivariable regression model that included both clinical and sociodemographic factors, enhancing the contextual relevance and robustness of the findings.

However, this study has important limitations. The absence of a baseline evaluation of the scales limits our ability to understand the changes that occurred after COVID-19 comprehensively. This, combined with the observational design and the single follow-up assessment, limits our ability to establish causality between COVID-19 and the observed psychiatric symptoms. Additionally, the inclusion of single-center patients further limits the generalizability and external validity of our results. Furthermore, the relatively small sample size may result in insufficient power to detect smaller effects. The population studied is very specific, as it included patients who suffered from severe COVID-19; therefore, the application of findings from this study should be approached with caution. The definition of severe COVID-19 used here is non-universal, as it was developed for CARDIO COVID 19–20. The absence of validated acute illness severity scores (such as APACHE-II and SAPS-II) and detailed ICU data, including sedative medication exposure, and major complications, limits both the clinical contextualization of our findings and a comprehensive understanding of the mechanisms underlying post-ICU cognitive outcomes. These unmeasured factors may act as important mediators or modifiers in the relationship between disease severity and long-term neuropsychiatric sequelae. The time between acute illness and this follow-up could contribute to a multifactorial effect on psychiatric abnormalities beyond the direct impact of COVID-19 and the pandemic. Finally, the use of low cutoff points for the PHQ-9 and GAD-7, although practical for detecting mild symptoms, could have inflated our prevalence estimates.

### Conclusion

Our findings reveal a high prevalence of mental health symptoms and cognitive dysfunction in patients 2 years after hospitalization for severe COVID-19. Interestingly, anxiety showed no differences by sex or ICU admission. In contrast, women had significantly higher rates of depression and perceived stress, while ICU admission emerged as an independent, severity-related factor associated with cognitive impairment. These patterns are likely multifactorial, potentially influenced by long COVID, pandemic-related stressors, and underlying multimorbidity. Our results underscore the need for systematic strategies to screen, diagnose, and manage mental health conditions in individuals with a history of COVID-19. However, due to the inherent limitations of our study, further confirmation from longitudinal and multicenter cohorts is warranted to better elucidate the long-term psychiatric and cognitive impact of COVID-19.

## Supplementary Material

Suppl 1 Occupational characteristics and socioeconomic strata by sex and ICU admission.

Suppl 2 Baseline characteristics stratified by sex and ICU admission.

## Data Availability

The datasets generated during and/or analyzed during the current study are available from the corresponding author on reasonable request.

## Abbreviations

ACE-III: Addenbrooke’s Cognitive Examination-III
ARB: angiotensin II receptor blocker
BMI: body mass index
CI: confidence interval
CIC: Centro de Investigaciones Clinicas
DBP: diastolic blood pressure
GAD-7: General Anxiety Disorder-7
ICU: intensive care unit
IQR: interquartile range
PHQ-9: Patient Health Questionnaire-9
PICS: post-intensive care syndrome
PSS-14: Perceived Stress Scale-14
PTSD: post-traumatic stress disorder
SBP: systolic blood pressure
SD: standard deviation
STROBE: Strengthening the Reporting of Observational Studies in Epidemiology
VIF: variance inflation factor
