# Supplementary material for: Long-Term Mental Health Evaluation After COVID-19: Insights From the CARDIO COVID 20–21 Registry
Source: J Clin Med Res. 2026 Jan 16;18(1):18–30. doi: 10.14740/jocmr6390 (PMC12861518; doi:10.14740/jocmr6390)
Supplement: Suppl 1 — Occupational characteristics and socioeconomic strata by sex and ICU admission. [file jocmr-18-01-018-s001.docx]

# **Supplementary Material**

**Suppl 1.** Occupational characteristics and socioeconomic strata by sex and ICU admission

| **Variable** | **All, N=152** | **Sex** | | | **ICU admission** | | |
| --- | --- | --- | --- | --- | --- | --- | --- |
|  |  | **Female, N=63** | **Male, N=89** | **p value** | **No, N=64** | **Yes, N=88** | **p value** |
| **Occupation** |  |  |  |  |  |  |  |
| Commercial | 63 (41%) | 26 (41%) | 37 (42%) | <0.001 | 28 (44%) | 35 (40%) | 0.016 |
| Unpaid work | 28 (18%) | 23 (37%) | 5 (5.6%) |  | 11 (17%) | 17 (19%) |  |
| Retired | 16 (11%) | 6 (9.5%) | 10 (11%) |  | 7 (11%) | 9 (10%) |  |
| Transportation | 13 (8.6%) | 1 (1.6%) | 12 (13%) |  | 1 (1.6%) | 12 (13%) |  |
| Health | 9 (5.9%) | 5 (7.9%) | 4 (4.5%) |  | 8 (13%) | 1 (1.1%) |  |
| Security | 5 (3.3%) | 1 (1.6%) | 4 (4.5%) |  | 1 (1.6%) | 4 (4.5%) |  |
| Agriculture | 5 (3.3%) | 1 (1.6%) | 4 (4.4%) |  | 1 (1.6%) | 4 (4.5%) |  |
| Construction | 5 (3.3%) | 0 (0%) | 5 (5.6%) |  | 3 (4.8%) | 2 (2.2%) |  |
| Legal services | 3 (2.0%) | 0 (0%) | 3 (3.3%) |  | 1 (1.6%) | 2 (2.2%) |  |
| Financial | 3 (2.0%) | 0 (0%) | 3 (3.3%) |  | 2 (3.2%) | 1 (1.1%) |  |
| Accounting | 1 (0.7%) | 0 (0%) | 1 (1.1%) |  | 0 (0%) | 1 (1.1%) |  |
| Entertaining/Art | 1 (0.7%) | 0 (0%) | 1 (1.1%) |  | 1 (1.6%) | 0 (0%) |  |
| **Socioeconomic strata^a^** |  |  |  |  |  |  |  |
| 1 | 33 (22%) | 17 (27%) | 16 (18%) | 0.7 | 15 (23%) | 18 (20%) | 0.2 |
| 2 | 49 (32%) | 19 (30%) | 30 (34%) |  | 17 (27%) | 32 (36%) |  |
| 3 | 36 (24%) | 13 (21%) | 23 (26%) |  | 13 (20%) | 23 (26%) |  |
| 4 | 13 (8.6%) | 5 (7.9%) | 8 (9.0%) |  | 5 (7.8%) | 8 (9.1%) |  |
| 5 | 16 (11%) | 8 (13%) | 8 (9.0%) |  | 11 (17%) | 5 (5.7%) |  |
| 6 | 5 (3.3%) | 1 (1.6%) | 4 (4.5%) |  | 3 (4.7%) | 2 (2.3%) |  |
| **Educational level** |  |  |  |  |  |  |  |
| Primary | 44 (29%) | 17 (27%) | 27 (30%) | 0.5 | 10 (16%) | 34 (39%) | 0.057 |
| Secondary | 47 (31%) | 22 (35%) | 25 (28%) |  | 23 (36%) | 24 (27%) |  |
| Technical | 16 (11%) | 6 (9.5%) | 10 (11%) |  | 8 (13%) | 8 (9.1%) |  |
| Technologist | 7 (4.6%) | 5 (7.9%) | 2 (2.2%) |  | 4 (6.3%) | 3 (3.4%) |  |
| Undergraduate | 25 (16%) | 10 (16%) | 15 (17%) |  | 14 (22%) | 11 (13%) |  |
| Postgraduate | 10 (6.6%) | 2 (3.2%) | 8 (9.0%) |  | 4 (6.3%) | 6 (6.8%) |  |
| No formal education | 3 (2.0%) | 1 (1.6%) | 2 (2.2%) |  | 1 (1.6%) | 2 (2.3%) |  |

*ICU: intensive care unit.*

*^a^In Colombia, residential areas are classified into six strata (1 to 6) based on income levels. Stratum 1 represents the lowest-income areas receiving the most subsidies for public utilities, and Stratum 6 represents the highest-income areas paying the highest rates with no subsidies.*
