# Supplementary material for: Long-Term Mental Health Evaluation After COVID-19: Insights From the CARDIO COVID 20–21 Registry
Source: J Clin Med Res. 2026 Jan 16;18(1):18–30. doi: 10.14740/jocmr6390 (PMC12861518; doi:10.14740/jocmr6390)
Supplement: Suppl 2 — Baseline characteristics stratified by sex and ICU admission. [file jocmr-18-01-018-s002.docx]

**Suppl 2.** Baseline characteristics stratified by sex and ICU admission

| **Variable** | **All, N=152** | **Sex** | |  | **ICU admission** | |  |
| --- | --- | --- | --- | --- | --- | --- | --- |
|  |  | **Female, N=63** | **Male, N=89** | **p value** | **No, N=64** | **Yes, N=88** | **p value** |
| **Comorbidities** |  |  |  |  |  |  |  |
| CKD | 14 (9.2%) | 8 (12%) | 7 (8%) | 0.4 | 7 (11%) | 8 (9%) | 0.7 |
| Asthma / COPD | 10 (6.6%) | 5 (7.9%) | 5 (5.7%) | 0.7 | 5 (7.9%) | 5 (5.7%) | 0.7 |
| Dyslipidemia | 9 (6.0%) | 1 (1.6%) | 8 (9.1%) | 0.08 | 4 (6.3%) | 5 (5.7%) | >0.9 |
| Cancer | 6 (4.0%) | 4 (6.3%) | 2 (2.3%) | 0.2 | 3 (4.8%) | 3 (3.4%) | 0.7 |
| Heart failure | 3 (1.9%) | 1 (1.6%) | 2 (2.3%) | >0.9 | 0 (0%) | 3 (3.4%) | 0.3 |
| Atrial fibrillation | 3 (1.9%) | 1 (1.6%) | 2 (2.3%) | >0.9 | 0 (0%) | 3 (3.4%) | 0.3 |
| **Pharmacological history** |  |  |  |  |  |  |  |
| Diuretic | 21 (14%) | 10 (16%) | 11 (12%) | 0.5 | 7 (11%) | 14 (16%) | 0.380 |
| Calcium channel blocker | 21 (14%) | 8 (13%) | 13 (15%) | 0.7 | 9 (14%) | 12 (14%) | 0.940 |
| Immunosuppressant | 20 (13%) | 11 (17%) | 9 (10%) | 0.2 | 13 (20%) | 7 (8.0%) | 0.026 |
| Beta-blocker | 19 (13%) | 6 (9.5%) | 13 (15%) | 0.4 | 5 (7.8%) | 14 (16%) | 0.136 |
| ACE inhibitor | 10 (6.6%) | 6 (9.5%) | 4 (4.5%) | 0.3 | 2 (3.1%) | 8 (9.1%) | 0.193 |
| Antiplatelet agent | 7 (4.6%) | 3 (4.8%) | 4 (4.5%) | >0.9 | 0 (0%) | 7 (8.0%) | 0.022 |
| Anticoagulant | 6 (3.9%) | 4 (6.3%) | 2 (2.2%) | 0.2 | 1 (1.6%) | 5 (5.7%) | 0.402 |
| Mineralocorticoid receptor antagonist | 6 (3.9%) | 2 (3.2%) | 4 (4.5%) | >0.9 | 1 (1.6%) | 5 (5.7%) | 0.402 |
| Sacubitril/valsartan | 3 (2.0%) | 0 (0%) | 3 (3.4%) | 0.3 | 1 (1.6%) | 2 (2.3%) | >0.999 |
| SGLT2 inhibitor | 3 (2.0%) | 1 (1.6%) | 2 (2.2%) | >0.9 | 0 (0%) | 3 (3.4%) | 0.264 |
| Biologic | 3 (2.0%) | 1 (1.6%) | 2 (2.2%) | >0.9 | 2 (3.1%) | 1 (1.1%) | 0.573 |
| Antiarrhythmic | 2 (1.3%) | 1 (1.6%) | 1 (1.1%) | >0.9 | 1 (1.6%) | 1 (1.1%) | >0.999 |

*Statistical significance was evaluated using Fisher’s exact test or Pearson’s Chi-squared test, as appropriate. ACE: Angiotensin-converting enzyme; CKD: C****hronic kidney disease;*** *COPD: Chronic obstructive pulmonary disease; ICU: Intensive care unit; SGLT2: Sodium–glucose cotransporter 2.*
